# Pasteurization temperatures effectively inactivate Rift Valley fever viruses in milk

**DOI:** 10.1128/jvi.02026-24

**Published:** 2025-01-30

**Authors:** Aurélie Pédarrieu, Camille Piro-Megy, Jordan Quellec, Catherine Cêtre-Sossah

**Affiliations:** 1 ASTRE, CIRAD, INRAE, University of Montpellier, Montpellier, France; St. Jude Children's Research Hospital, Memphis, Tennessee, USA

**Keywords:** Rift Valley fever virus, epidemiology, inactivation, milk

## LETTER

Rift Valley fever virus (RVFV) is a zoonotic arbovirus widely distributed across Africa, the Arabian Peninsula, and islands of the South West of the Indian Ocean, which causes recurrent epidemics affecting ruminant livestock as well as humans. RVFV is transmitted to humans by two main routes of infection, indirectly by infected vectors or directly by viral aerosolization from infected livestock tissues and body fluids (1).

The mode of transmission involved in recent RVF outbreaks in Kenya or Mayotte was not well identified for all cases, but the most commonly reported were exposure to animals or consumption of raw milk (2, 3). International agencies continue to seek RVF experts’ recommendations and opinions on the best applicable control and prevention measures (4). To date, no studies have addressed the efficacy of temperature inactivation methods for infectious RVFV in milk. Pasteurization is a well-established method of heat inactivation, initially formalized by Pasteur for wine in 1864 (5).

To assess the effects of pasteurization on RVFVs in milk and its usefulness in preventive and sanitary measures against RVF, we spiked whole cow’s milk with two RVFV viral strains, the Smithburn vaccine strain (6) or the MRU 25010-30 RVFV virulent field strain (7). Two temperature conditions representing the two common methods of pasteurizing milk were tested: (i) low-temperature long time (LTLT), which requires heating to 62.5°C for at least 30 minutes (8); and (ii) high-temperature short time (HTST), which requires heating to 72°C for at least 15 seconds (9). Vero cells grown at 37°C–5% CO_2_ in minimal essential medium (MEM, Gibco, USA) supplemented with 2 mM L-glutamine and 10% decomplemented fetal bovine serum (FBS) (Corning, France) were infected in triplicate at a multiplicity of infection (MOI) of 0.1 in a 96-well plate format (1 × 10^4^ cells/well). Inocula, prepared by diluting the virus from the viral stock in whole cow’s milk, were pasteurized using both mentioned methods and added to Vero cells at 37°C for 60 minutes. The medium was then replaced by complete MEM and incubated for 5 days. A mock control, consisting of the inoculum with medium only, was included.

**Fig 1:**
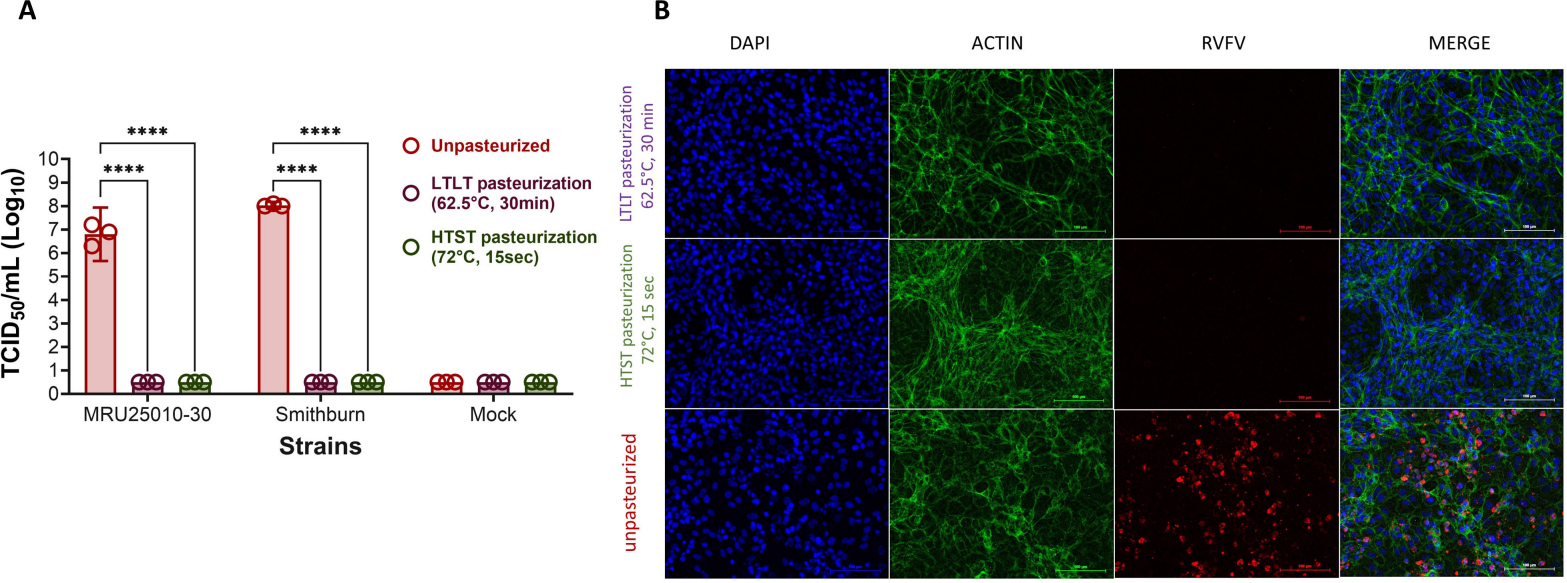
Pasteurization temperatures inactivate RVFVs in milk. (A) Viral quantification was measured using TCID_50_ method in mock and RVFV-infected supernatants (Smithburn and MRU 25010–30, MOI 0.1) harvested 5 days post-infection (dpi). Two-way analysis of variance (ANOVA) (****P < 0.0001). (B) RVFV-infected cells (Smithburn and MRU 25010–30, MOI 0.1) were fixed at 5 dpi using a 4% paraformaldehyde (PFA) solution for 15 minutes at room temperature (RT) and were labeled with a mouse monoclonal IgG1 anti-nucleoprotein N RVFV antibody (anti-N RVFV, 1:50 [Cirad, 15G6-4B8]) and a secondary antibody (polyclonal donkey IgG anti-mouse IgG Alexa Fluor 555 [1:500 {Invitrogen A31570}], red), an actin probe (Actin Green [Ready Probes, Alexa Fluor 488, 1:50 {Thermo Fisher, 37110}, green], and DAPI (1:1,000 [Sigma-Aldrich, MBD0015], blue) for nuclear labeling (scale bar, 100 µm). Labeled wells were imaged by inversed microscope AXIOVERT A1 (Zeiss, France) using Archimed 6.1.4 software and were analyzed with Image J Software bundled with 64-bit Java 1.8.0_172. Similar data were obtained for both strains, only Smithburn-infected conditions are presented.

We first evaluated RVFV infection by quantifying viral titers by tissue culture infectious dose 50% (TCID_50_) calculated with Spearman-Kärber method (10) in infected Vero supernatants. We showed a significant decrease in viral titers to the limit of detection of the method for both strains in milk heated using both methods of pasteurization (Fig. 1A). We next confirmed these findings in milk-infected Vero cells with RVFV-specific labeling (Fig. 1B) (antibody anti-RVFV N nucleoprotein, red) that was only present in unpasteurized conditions and absent in pasteurized conditions.

These results demonstrate that commonly performed pasteurization techniques fully inactivate RVFV in milk, which is an important information to share considering the widespread public health risk that raw milk consumption represents in the RVF transmission cycle. This study provides baseline evidence to guide and strengthen future safety recommendations to avoid RVFV transmission through unpasteurized raw milk contributing to the spread of RVFV outbreaks.

### Biosafety statement

All experiments were conducted in biosafety level 3 (BSL-3) laboratories while dealing with infectious materials or biosafety level 2 (BSL-2) laboratories when working on inactivated materials.
